# Beyond the LARS score: functional burden does not necessarily equal psychological burden

**DOI:** 10.1007/s00384-026-05261-0

**Published:** 2026-11-05

**Authors:** Serdar Gumus

**Affiliations:** Surgical Oncology, Cukurova University, Faculty of Medicine, Adana, Turkey

**Keywords:** Low anterior resection syndrome, Patient-reported outcomes, Quality of life, Psychological burden, Resilience, Rectal cancer

## Abstract

**Purpose:**

Ammer et al. reported no significant difference in low anterior resection syndrome (LARS) between open and robotic rectal cancer surgery. This commentary addresses the interindividual variability in LARS outcomes.

**Methods:**

This conceptual commentary critically reviews the outcome assessment strategy used in the original study and integrates supporting evidence from the psycho-oncology literature.

**Results:**

Functional burden, as captured by the LARS score, does not necessarily equal psychological burden. Psychological adaptation—resilience, coping style, distress, and illness perception—is hypothesised to mediate the relationship between functional impairment and patient-reported quality of life.

**Conclusion:**

Functional burden and psychological burden are related but distinct constructs. Future studies should test mediation and moderation models to determine whether psychological adaptation explains the variability between LARS severity and quality of life.

I read with great interest the recent article by Ammer et al. comparing the incidence and severity of low anterior resection syndrome (LARS) after open versus robotic surgery using a patient-reported outcome measure (PROM) [1]. I commend the authors for an honest and clinically important negative finding—that robotic surgery does not appear to be inherently protective against LARS—which has direct implications for preoperative counselling.

I would like to offer a different lens on this negative finding, one centred on a distinction I believe the wider LARS literature has yet to make explicit: functional burden does not necessarily equal psychological burden. The LARS score is, by design, a measure of bowel dysfunction—frequency, urgency, incontinence, clustering. It was never intended to capture how a given patient experiences that dysfunction psychologically. Two patients with identical functional impairment may experience profoundly different psychological burden, depending on their resilience, coping style, illness perception, and baseline distress. I would argue that it is this psychological layer—not the functional score itself—that may substantially influence patient-reported quality of life.

This distinction is not merely theoretical. In a cohort of 105 rectal cancer patients, Lee and Kim found that anxiety and depression predicted quality of life independently of LARS severity [2]. The MANUEL project already implicitly acknowledges this distinction: it recommends supplementing the LARS score with a broader instrument such as the EORTC QLQ-C30 because psychosocial late effects of rectal cancer treatment are not captured by bowel symptom scores alone [3]. Ammer et al.’s reliance on a single-item proxy for quality of life, rather than a validated multidimensional instrument, further illustrates this broader methodological challenge [1].

A recent analysis of 115 patients undergoing low anterior resection found no significant difference in LARS severity across open, laparoscopic, and robotic approaches, whereas low anastomotic height showed a substantially stronger association with LARS [4]. A multicentre randomised trial comparing laparoscopic and transanal total mesorectal excision likewise reported comparable LARS scores and EORTC QLQ-C30 global health status between the two approaches, despite differences in individual bowel symptoms [5]. Taken together, these findings suggest that surgical approach alone is unlikely to account for the substantial variability in LARS-related outcomes. I propose that psychological adaptation may represent one of these missing links: psychological adaptation may act as a bridge between bowel dysfunction and quality of life. A patient’s LARS score explains only part of their outcome; the remainder may partly reflect how that patient processes, interprets, and copes with the dysfunction.

I therefore propose the following conceptual pathway, which reframes the relationship between surgery, symptoms, and outcome (Fig. 1):

**Fig. 1:**
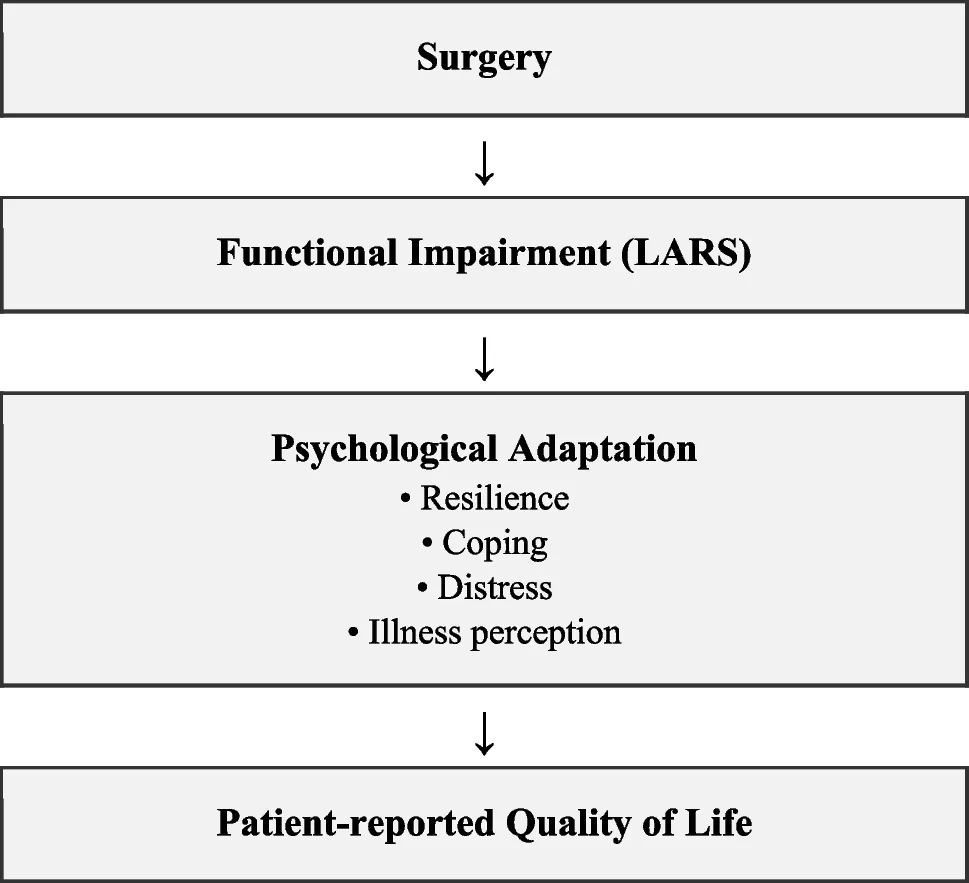
Proposed conceptual model of LARS-related outcomes. Functional impairment following rectal cancer surgery is filtered through psychological adaptation—resilience, coping, distress, and illness perception—before potentially influencing patient-reported quality of life. Functional burden and psychological burden are related but distinct constructs

The more fundamental question becomes why patients with comparable functional impairment diverge so widely in psychological and quality-of-life outcomes. Future studies should explore mediation and moderation models to determine whether psychological adaptation explains the variability between symptom severity and patient-reported quality of life, rather than treating LARS score and quality of life as directly and exclusively linked. Concretely, this could be operationalised by pairing the LARS score with validated instruments assessing resilience (e.g., Connor-Davidson Resilience Scale), coping style (e.g., Brief COPE), and illness perception (e.g., Brief Illness Perception Questionnaire).

Such a reframing would not diminish the value of the present findings but would extend them: if functional burden does not necessarily equal psychological burden, then surgical approach—whichever form it takes—is unlikely to fully explain how patients ultimately fare. This conceptual model has not yet been empirically tested, and its central claim—that psychological adaptation mediates the relationship between LARS severity and quality of life—should for now be read as a hypothesis rather than an established finding. Even so, the next advance in LARS research may depend less on further refining the measurement of bowel function than on understanding why patients with similar functional impairment experience fundamentally different psychological outcomes.

## Data Availability

No datasets were generated or analysed during the current study.
